# The Pepper CaOSR1 Protein Regulates the Osmotic Stress Response via Abscisic Acid Signaling

**DOI:** 10.3389/fpls.2016.00890

**Published:** 2016-06-24

**Authors:** Chanmi Park, Chae Woo Lim, Sung Chul Lee

**Affiliations:** Department of Life Science (BK21 Program), Chung-Ang UniversitySeoul, South Korea

**Keywords:** abscisic acid, *CaOSR1*, drought stress, osmotic stress, pepper, virus-induced gene silencing

## Abstract

Plants are sessile organisms, and their growth and development is detrimentally affected by environmental stresses such as drought and high salinity. Defense mechanisms are tightly regulated and complex processes, which respond to changing environmental conditions; however, the precise mechanisms that function under adverse conditions remain unclear. Here, we report the identification and functional characterization of the *CaOSR1* gene, which functions in the adaptive response to abiotic stress. We found that *CaOSR1* gene expression in pepper leaves was up-regulated after exposure to abscisic acid (ABA), drought, and high salinity. In addition, we demonstrated that the fusion protein of CaOSR1 with green fluorescent protein (GFP) is localized in the nucleus. We used *CaOSR1*-silenced pepper plants and *CaOSR1*-OX-overexpressing (OX) transgenic Arabidopsis plants to show that the CaOSR1 protein regulates the osmotic stress response. *CaOSR1*-silenced pepper plants showed increased drought susceptibility, and this was accompanied by a high transpiration rate. *CaOSR1*-OX plants displayed phenotypes that were hypersensitive to ABA and hyposensitive to osmotic stress, during the seed germination and seedling growth stages; furthermore, these plants exhibited enhanced drought tolerance at the adult stage, and this was characterized by higher leaf temperatures and smaller stomatal apertures because of ABA hypersensitivity. Taken together, our data indicate that *CaOSR1* positively regulates osmotic stress tolerance via ABA-mediated cell signaling. These findings suggest an involvement of a novel protein in ABA and osmotic stress signalings in plants.

## Introduction

Drought, cold, and high salinity in the soil cause osmotic stress to plants, and limit plant growth and development and agricultural crop productivity (Zhu, 2002; Sengupta and Majumder, 2010). In the natural environment, growth conditions are rarely optimal and plants constantly encounter various osmotic stresses; these alter the water potential in plant cells and cause serious damage (Grondin et al., 2015). During exposure to osmotic stresses, plants exhibit many physiological and molecular changes such as reduction of water content, stomatal closure, alteration of cell growth, and inhibition of photosynthesis, to limit cellular damage and restore homeostasis (Zhu, 2002; Ambrosone et al., 2015). The cellular and physiological defense-related response to osmotic stress has been elucidated; however, the precise mechanisms underlying functional modification remain unclear, because of the complexity and diversity at the cellular level and the whole plant level (Ding et al., 2015; Lim et al., 2015a; Zou et al., 2015).

Abscisic acid (ABA) is a plant hormone that promotes the processes of adaptation to abiotic and biotic stresses (Gonzalez-Guzman et al., 2012; Lee and Luan, 2012; Lim et al., 2015a). ABA plays a primary role in the cellular defense response under osmotic stress conditions. The level of ABA is increased in response to osmotic stress, and this initiates the signal transduction pathway related to defense responses. In comparison with other plant hormones, ABA regulates a large number of genes; more than 10% of Arabidopsis genes are induced by ABA (Goda et al., 2008; Mizuno and Yamashino, 2008). The most well-known response is ABA-mediated stomatal closure via the efflux of cations and anions from guard cells; this leads to decreased transpiration, which is vital for plant survival under osmotic stress conditions (Geiger et al., 2009; Lee et al., 2009). Hence, ABA-responsive and ABA-deficient mutants exhibit a phenotype that is tolerant to abiotic stress (Vlad et al., 2009; Umezawa et al., 2013; Zou et al., 2015). In contrast, ABA hypersensitivity is associated with abiotic stress tolerance (Lee et al., 2013; Lim and Lee, 2015). Moreover, a large number of genes associated with the defense response to osmotic stress are regulated by ABA (Shinozaki and Yamaguchi-Shinozaki, 2007; Lim et al., 2015b). Genetic and molecular analysis studies have identified many stress-related genes and transcription factors involved in defense responses. For example, ABA signal transduction components—from ABA receptors to SnRK2-type kinase—regulate ion channels and bZIP transcription factors (Lee and Luan, 2012; Lim et al., 2015a). In addition, transcription factors—including dehydration-responsive element binding protein/c-repeat binding factor (DREB/CBF) (Lee et al., 2010; Lee and Thomashow, 2012; Ding et al., 2015), ABA binding factor (ABF) (Kim et al., 2004; Yoshida et al., 2015), and ethylene-responsive factor/Apetala2 (ERF/AP2) (Li et al., 2011; Dubois et al., 2015)—activate different defense-related genes in response to osmotic stress, to be rebalanced and fine-tuned.

In the present study, we identified a novel pepper gene, *CaOSR1* (*C**apsicum* *a**nnuum* Osmotic Stress Resistance 1). We isolated this gene from a pepper cDNA library, using an ABA-inducible cDNA as a probe. We examined the molecular and phenotypic aspects of *CaOSR1*-silenced pepper and *CaOSR1*-overexpressing (OX) transgenic Arabidopsis plants. We showed that the alteration of *CaOSR1* expression influences drought stress tolerance via regulation of the transpiration rate and induction of stress-responsive genes. Our findings indicate that the CaOSR1 protein is a positive regulator of ABA signaling and osmotic stress tolerance.

## Materials and methods

### Plant materials

Seeds of pepper (*Capsicum annuum* L., cv. Nockwang) and tobacco (*Nicotiana benthamiana*) were sown in a steam-sterilized compost soil mix (peat moss, perlite, and vermiculite, 5:3:2, v/v/v), sand, and loam soil (1:1:1, v/v/v). The pepper plants were raised in a growth room at 27 ± 1°C under white fluorescent light (80 μmol photons·m^−2^·s^−1^; 16 h per day) as described previously (Lee et al., 2008). The tobacco plants were maintained in a growth chamber at 25 ± 1°C under a 16-h light/8-h dark cycle. *Arabidopsis thaliana* (ecotype Col-0) seeds were germinated on Murashige and Skoog (MS) salt (Duchefa Biochemie) supplemented with 1% sucrose and Microagar (Duchefa Biochemie); the seeded plates were incubated in a growth chamber at 24°C under a 16-h light/8-h dark cycle. The Arabidopsis seedlings were maintained in a steam-sterilized compost soil mix (peat moss, perlite, and vermiculite, 9:1:1, v/v/v) under controlled environmental conditions as follows: 24°C and 60% relative humidity under fluorescent light (130 μmol photons·m^−2^·s^−1^) with a 16-h light/8-h dark cycle. All seeds were vernalized at 4°C for 2 days before being placed in the growth chamber.

### Sequence alignment and phylogenetic tree analysis

The encoded amino acid sequences for CaOSR1 and its homologs were obtained using BLAST searches (http://www.ncbi.nlm.nih.gov/BLAST). The amino acid alignment was performed using ClustalW2 (http://www.ebi.ac.uk/Tools/msa/clustalw2), and the results were edited using Genedoc software (http://www.nrbsc.org/gfx/genedoc). The amino acid alignments were manually regulated to compare the cDNA clones of CaOSR1 with those of other organisms. Based on the data of multiple sequence alignment, phylogenetic tree was drawn with MEGA software (version 5.2). To investigate sequence identity and similarity between two proteins, pairwise sequence alignment was performed using EMBOSS Needle webtool (http://www.ebi.ac.uk/Tools/psa/emboss_needle) with default parameter.

### Virus-induced gene silencing and overexpression of *CaOSR1*

We used the tobacco rattle virus (TRV)-based virus-induced gene silencing (VIGS) system to generate *CaOSR1* gene knockdown in pepper plants. We used a 1598–1805-bp fragment and the full length *CaOSR1* cDNA to generate *CaOSR1*-silenced pepper plants and *CaOSR1*-overexpressing (OX) transgenic Arabidopsis plants, respectively, according to the protocol described previously (Park C. et al., 2015).

### ABA, drought, and NaCl treatments

To examine the expression pattern of the *CaOSR1* gene in pepper plants after ABA treatment, six-leaf-stage pepper plants were sprayed with 100 μM ABA or control solution. For the NaCl and drought treatments, pepper plants were irrigated with 200 mM NaCl solution and were then carefully removed from the soil to avoid injury. The plants were placed onto 3-mm filter paper (Whatman, Clifton, UK). Leaves were harvested at 0–24 h after each treatment and were subjected to RNA isolation and reverse transcription-polymerase chain reaction (RT-PCR) analysis.

To measure the rate of germination, root elongation and seedling establishment, 36 seeds each of wild-type and *CaOSR1*-OX transgenic Arabidopsis plants were stratified at 4°C for 2 days and were then plated on 0.5 × MS agar medium supplemented with various concentrations of ABA. The plates were incubated at 24°C under white fluorescent light (130 μmol photons·m^−2^·s^−1^) with a 16-h light/8 h-dark cycle.

Three-week-old seedlings from wild-type and *CaOSR1*-OX transgenic Arabidopsis lines were randomly planted and were then subjected to drought stress treatment by withholding watering for 9 days and rewatering for 2 days. Survival rates were measured in each individual sample, and each experiment was performed three times with 20 plants. For pepper plants, drought stress was imposed on four-leaf-stage plants by withholding watering for 12 days. Plants were rewatered for 2 days to allow recovery, and the survival rate of the plants was then calculated. Survival rates were measured in each individual sample, and each experiment was performed three times with 20 plants. The drought resistance was determined in a quantitative manner by measuring the transpirational water loss. Fifty leaves were detached from four-leaf-stage pepper plants and 3-week old Arabidopsis plants and placed in Petri dishes. The dishes were maintained in a growth chamber at 40% relative humidity, and the loss of fresh weight was determined at the indicated time points. All the experiments were performed at least in triplicate.

### Thermal imaging

For thermal imaging analysis, 4-week-old pepper plants having full expanded 1st and 2nd leaves and 3-week-old Arabidopsis plants and were treated with 50 μM ABA. Thermal images were obtained using an infrared camera (FLIR systems; T420) and leaf temperature was measured by FLIR Tools + ver 5.2 software.

### Stomatal aperture bioassay

To measure the stomatal aperture, epidermal peels were stripped from rosette leaves of 3-week-old plants and floated in a stomatal opening solution (SOS: 50 mM KCl and 10 mM MES-KOH, pH 6.15, 10 μM CaCl_2_) in the light. After incubation for 3 h, the buffer was replaced with fresh SOS containing 20 μM ABA. After additional 2 h incubation, stomatal apertures were measured in each individual sample, and each experiment was performed three times with 20 leaves.

### RNA isolation and semi-quantitative and quantitative reverse transcription-polymerase chain reaction

Total RNA was isolated from the Arabidopsis leaf tissues, which were dehydrated or infected with the bacterial pathogen using an RNeasy Mini kit (Qiagen, Valencia, CA, USA). To remove genomic DNA, all RNA samples were digested with RNA-free DNase. After quantification using a spectrophotometer, 1 μg of total RNA was used to synthesize cDNA using a Transcript First Strand cDNA Synthesis kit (Roche, Indianapolis, IN, USA) according to the manufacturer's instructions. Concomitantly, cDNAs were synthesized without reverse transcriptase and were subjected to semi-quantitative RT-PCR to rule out the possibility of contamination by genomic DNA in the cDNA samples. For quantitative reverse transcription-polymerase chain reaction (qRT-PCR) analysis, the synthesized cDNA was amplified in a CFX96 Touch™ Real-Time PCR detection system (Bio-Rad) with iQ™SYBR Green Supermix and specific primers (Supplementary Table 1). Every reaction was performed in triplicate. The PCR was programmed as follows: 95°C for 5 min; 45 cycles each at 95°C for 20 s and 60°C for 20 s; and 72°C for 20 s. The relative expression of each gene was calculated using the ΔΔCt method, as previously described (Livak and Schmittgen, 2001). The Arabidopsis *actin8* gene (*AtACT8*) was used for normalization.

### Statistical analyses

To determine significant differences between genotypes in response to treatments, statistical analyses were performed using one way analysis of variance (ANOVA) or student's *t*-test. A *P* < 0.05 was considered significant difference.

## Results

### Isolation and sequence analysis of the pepper *CaOSR1* gene

**Figure 1:**
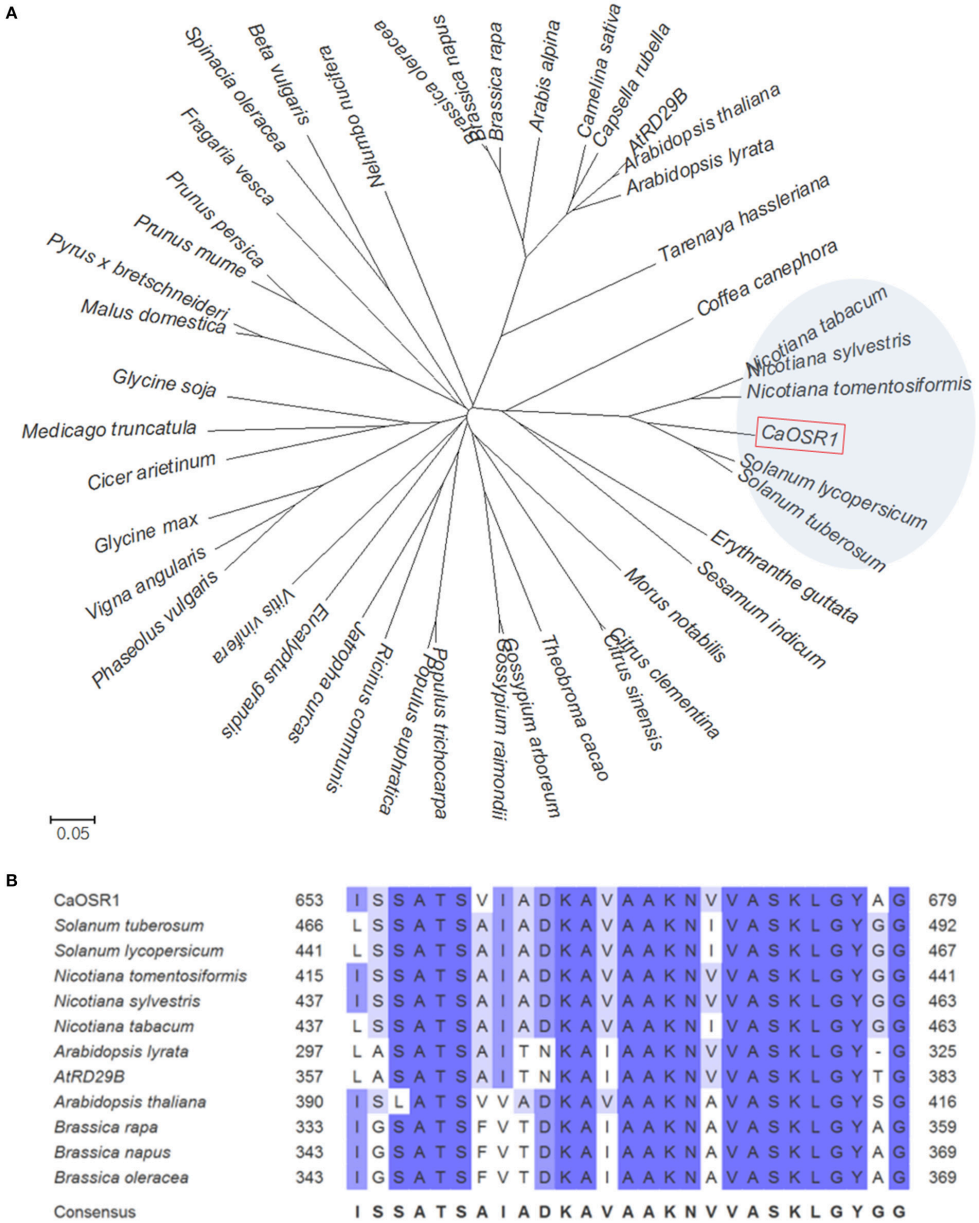
**Homology of the pepper CaOSR1 (***C******apsicum******a******nnuum*****O**smotic **S**tress **R**esistance 1) protein with low-temperature induced proteins**. **(A)** Phylogenetic tree analysis of CaOSR1 protein. Blast search was performed by using deduced amino acid sequences of *CaOSR1* gene and top ranked sequences from each plant species were gathered. For Arabidopsis, as CaOSR1 homolog, a well-known gene RD29B (At5g52300; accession number BAA02375) and it's homologous gene CAP160 (At4g25580; accession number NP_194288) were also added. Multiple alignment of those amino acid sequences was performed by using ClustalW2 and the phylogenetic tree was drawn with MEGA software (version 5.2). **(B)** Multiple sequence alignment analysis of Cold Acclimation Protein (CAP) 160 domain. Amino acid residues of CAP160 domains are shaded according to percent identity in ClustalW.

We used differential hybridization analysis to isolate the pepper *CaOSR1* (C*apsicum* a*nnuum* Osmotic Stress Resistance 1) gene from a cDNA library constructed from ABA-treated pepper leaf tissues (Lim et al., 2014). Among the gene clones, we selected genes that were upregulated by ABA (data not shown). The putative *CaOSR1* consists of a 2643-bp open reading frame, and the predicted *CaOSR1* encodes 880 amino acid residues (Figure 1) The mature protein has a molecular weight of 93,865 Da and an isoelectric point of 5.65. The results of multiple sequence alignment analysis and the phylogenetic tree showed that CaOSR1 (accession no. KT693385) is clustered into the same clade with five low-temperature-induced 65 kDa (LTI65) proteins from the family *Solanaceae* (Figure 1A) of which LTI65 protein of *Solanum tuberosum* (accession no. XP_006353392.1) shares highest identity (60.9%) and similarity (64.3%) withCaOSR1. Although sequence homology is < 35%, CaOSR1 shares sequence homology with three proteins from Arabidopsis such as RD29B (LTI65; At5g52300), CAP160 (At4g25580), and RD29A (COR78; At5g52310). In particular, RD29B shares 26.4% identity and 34.3% similarity with CaOSR1. Domain analysis revealed that CaOSR1 have two conserved regions: acidic region (72–88 aa) and CAP160 domain (653–679 aa), which are shown in common in most of CaOSR1-homologous proteins (Figure 1B and Supplementary Figure 1). CAP160 domain is first reported in spinach cold acclimation protein (CAP) 160 protein (Kaye et al., 1998). Although the precise function of CAP160 is still unknown, CAP160 is induced by drought stress as well as low temperature exposure (Kaye et al., 1998). These stresses involve induction of dehydration and plants respond to them very similarly at molecular level (Shinozaki and Yamaguchi-Shinozaki, 2000). Based on the data, we proposed that CaOSR1 may function in plant response to dehydration-involved stresses.

### Induction of the *CaOSR1* gene by ABA, drought, and high salt stress treatments and subcellular localization of the CaOSR1 protein

**Figure 2:**
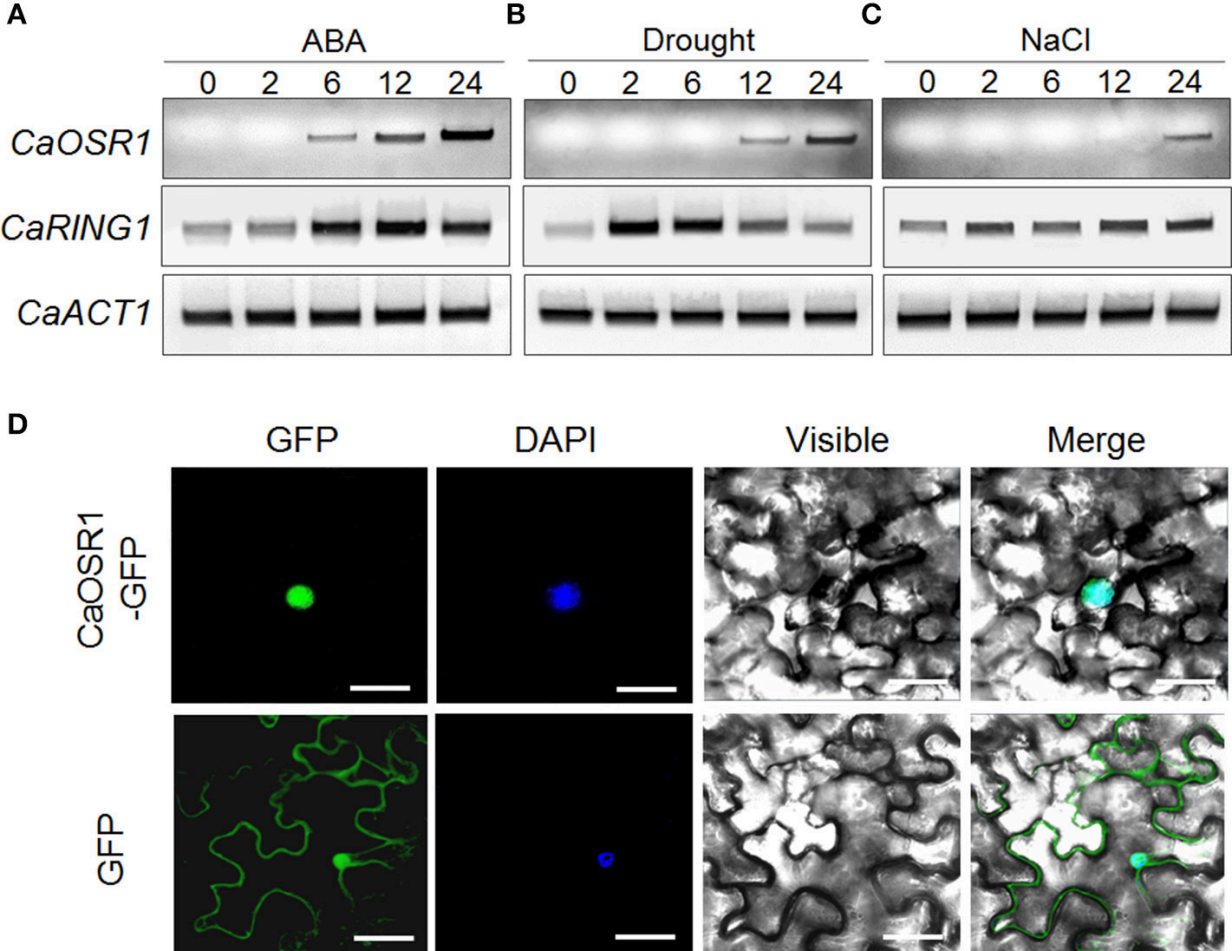
**Expression of the ***CaOSR1*** gene and localization of the CaOSR1 protein**. Induction of the *CaOSR1* gene in pepper leaves at various time points after treatment with 100 μM abscisic acid (ABA) **(A)**, drought **(B)**, or 200 mM NaCl **(C)**. The pepper *CaRING1* and *Actin1* genes were used as experimental and internal controls, respectively. **(D)** Subcellular localization of the CaOSR1 protein using transient expression of the green fluorescent protein (GFP) fusion protein in *Nicotiana benthamiana* cells. The 35S:*CaOSR1-GFP* construct was expressed using agroinfiltration of *N. benthamiana* leaves and observed under a confocal laser-scanning microscope. 4′,6-Diamidino-2-phenylindole (DAPI) staining was used as a marker for the nucleus. White bar = 10 μm.

The *CaOSR1* gene was isolated from ABA-treated pepper leaves, using the differential hybridization assay (Lim et al., 2014). To investigate the potential involvement of *CaOSR1* in dehydration stress responses, we examined the expression levels of this gene after ABA, drought, and high salinity treatments (Figure 2). We found that the accumulation of *CaOSR1* transcripts was first detected at 6 h after ABA treatment and reached a maximum level at 24 h (Figure 2A). ABA and abiotic stress signals, including drought and high salt, seem to share common elements in their respective signaling pathways; however, these stress signals are not solely dependent on ABA signaling (Jakab et al., 2005). In the present study, we found that after drought stress treatment, the accumulation of *CaOSR1* transcripts was first detected at 12 h and reached a maximum level at 24 h (Figure 2B). On the other hand, after treatment with NaCl, *CaOSR1* transcripts were weakly expressed at 24 h (Figure 2C). Our data suggest that *CaOSR1* functions in osmotic stress responses.

To investigate the subcellular localization of the CaOSR1 protein in plant cells, we fused the green fluorescent protein (GFP) reporter gene to the C-terminal region of *CaOSR1* under the control of the 35S promoter. We found that expression of the 35S:*CaOSR1-GFP* fusion protein in *Nicotiana benthamiana* epidermal cells generated GFP signals in the nucleus (Figure 2D). We used DAPI staining as a nucleus marker, and observed that the blue signals localized to nucleus overlapped with the GFP signals.

### Increased susceptibility of *CaOSR1*-silenced pepper plants to drought stress

**Figure 3:**
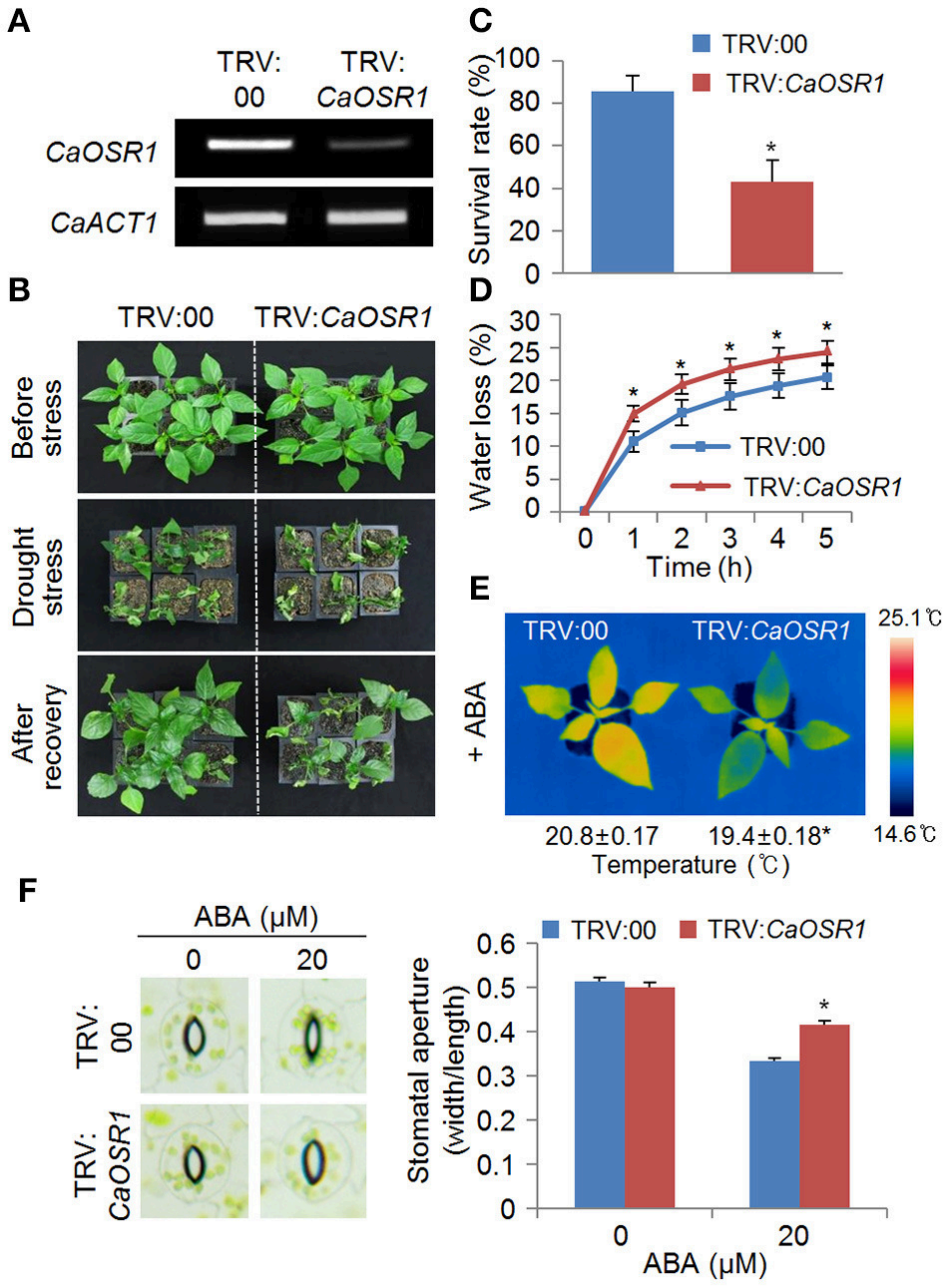
**Increased susceptibility of ***CaOSR1***-silenced pepper plants to drought stress**. **(A)** Reverse transcription-polymerase chain reaction (RT-PCR) analysis of *CaOSR1* gene expression in the leaves of pepper plants transfected with the empty vector control (TRV:00) or *CaOSR1*-silenced constructs (TRV:*CaOSR1*-RNAi) 0 h after detachment. The *Actin1* gene was used as an internal control. **(B)** Drought susceptibility of *CaOSR1*-silenced pepper plants. Empty vector control and *CaOSR1* gene-silenced pepper plants were grown in pots for 5 weeks under normal growth conditions. The plants were then subjected to drought stress by withholding watering for 12 days, followed by rewatering for 2 days. Representative images were taken before (upper) and after (middle) dehydration, and 2 days after rewatering (lower). **(C)** The survival rate was measured by counting the number of plants with green and rehydrated leaves 2 days after rewatering. **(D)** Transpirational water loss from the leaves of empty vector control and *CaOSR1* gene-silenced pepper plants at various times after detachment of leaves. Data represent the mean ± standard deviation of three independent experiments. **(E)** Decreased leaf temperatures of *CaOSR1* gene-silenced pepper plants after ABA treatment. Data represent the mean ± standard error of three independent experiments. **(F)** Stomatal apertures in empty vector control and *CaOSR1* gene-silenced pepper plants after ABA treatment. Leaf peels were harvested from 2-week-old pepper plants and incubated in stomatal opening solution (SOS) buffer containing 20 μM ABA; the stomatal apertures were then measured under a microscope. Representative images were taken before (left) and after (right) 2 h of ABA treatment. Data represent the mean ± standard error of three independent experiments, each evaluating 20 plants. Asterisks indicate significant differences between the control and the *CaOSR1*-silenced pepper plants (Student's *t*-test; *P* < 0.05).

The *CaOSR1* gene was induced by abiotic stresses, and therefore we postulated that *CaOSR1* is involved in osmotic stress responses. To test this hypothesis, we performed VIGS-based gene function analysis in pepper plants and an overexpression assay in Arabidopsis plants. We examined the level of VIGS using reverse transcription-polymerase chain reaction (RT-PCR) analysis of control (TRV:00) and *CaOSR1*-silenced pepper (TRV:*CaOSR1*-RNAi) leaves. We found that *CaOSR1* expression in the leaves of *CaOSR1*-silenced peppers was compromised but remained marginally detectable (Figure 3A). The expression of *CaOSR1* was induced by drought stress, implying that this gene functions in the drought stress response. Hence, we compared the phenotypes displayed by *CaOSR1*-silenced pepper plants and control plants after drought stress treatment (Figure 3B). Under well-watered conditions, we observed no phenotypic differences between control and *CaAIP1*-silenced pepper plants (Figure 3B, upper panel). However, when we subjected plants to drought stress by withholding watering for 12 days, *CaOSR1*-silenced pepper plants displayed a more wilted phenotype than control plants (Figure 3B, middle panel). After recovery by rewatering, *CaOSR1*-silenced pepper plants resumed growth more slowly than control plants (Figure 3B, lower panel). Moreover, the survival rates of *CaOSR1*-silenced plants and control plants were 42.8 and 85.7%, respectively (Figure 3C). Based on the wilted phenotype displayed by *CaOSR1*-silenced pepper plants after drought stress treatment, we postulated that transpirational water loss in detached *CaOSR1*-silenced pepper leaves is increased because of reduced water retention. To test this hypothesis, we analyzed the transpiration rate in the leaves of control plants and *CaOSR1*-silenced pepper plants. We found that the rate of water loss was higher in the leaves of *CaOSR1*-silenced pepper plants than in the leaves of control plants (Figure 3D). The observed phenotypic discrepancy between *CaOSR1*-silenced pepper plants and control plants under drought stress conditions prompted us to investigate the ABA sensitivity by measuring the leaf temperatures and stomatal apertures. The leaf temperature decreases when the stomata open, because of evaporative cooling. We found that before ABA treatment, the leaf temperatures are not significantly different between both plants (Supplementary Figure 2A). However, the leaf temperatures of *CaOSR1*-silenced pepper plants were significantly lower than those of control plants after ABA treatment (Figure 3E). The ABA sensitivity can further be determined by measuring the change in stomatal pore size in response to ABA treatment. In the absence of ABA, we determined no significant differences in stomatal aperture between the leaves of control plants and *CaOSR1*-silenced pepper plants (Figure 3F). However, consistent with our leaf temperature data, we found that after ABA treatment, the stomatal apertures were significantly larger in the leaves of *CaOSR1*-silenced pepper plants than in the leaves of control plants. Taken together, our results imply that the high rate of transpiration, and hence the increased drought susceptibility of *CaOSR1*-silenced pepper plants, is mainly derived from reduced ABA sensitivity.

### Enhanced sensitivity of *CaOSR1*-OX transgenic plants to ABA

**Figure 4:**
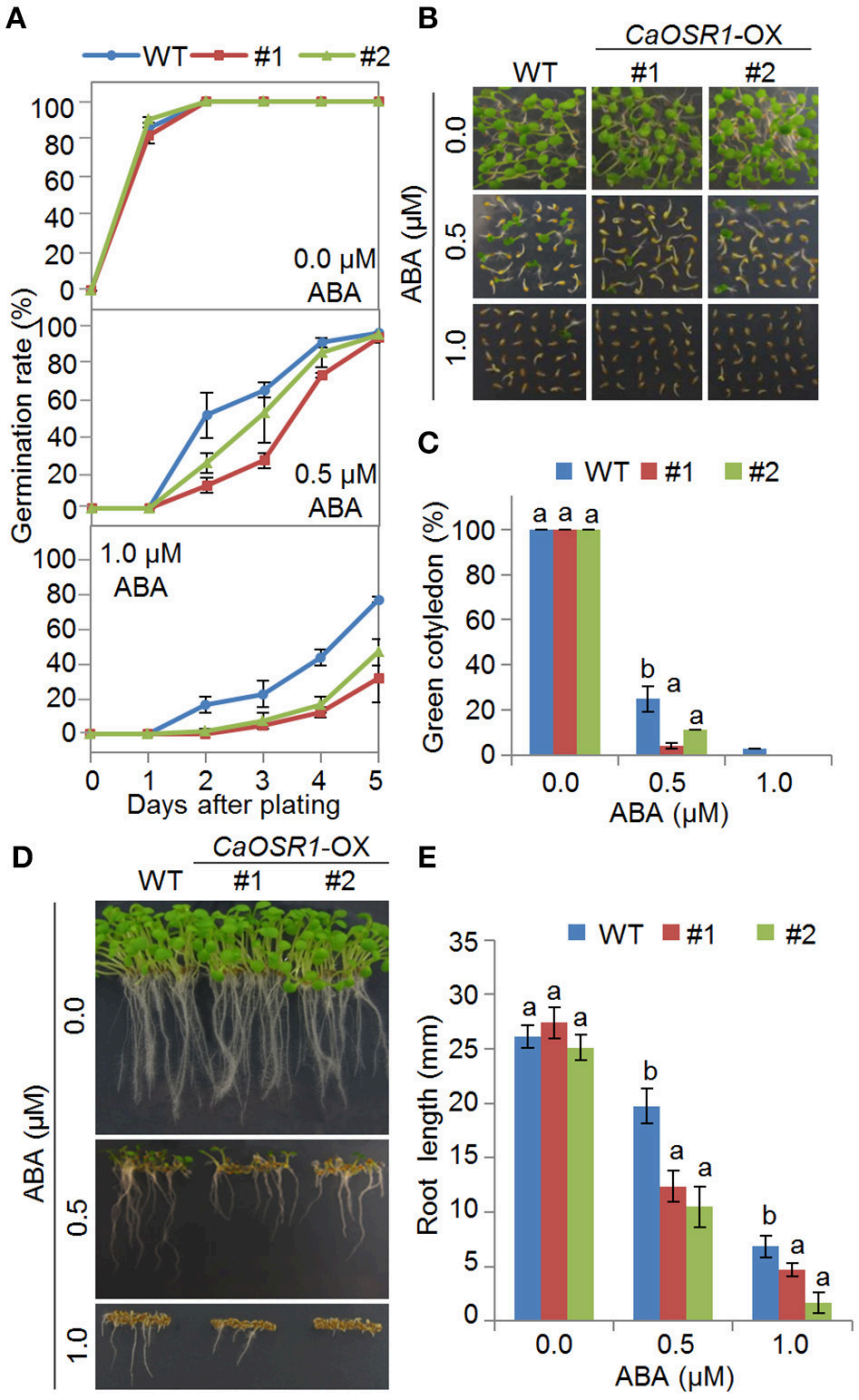
**Enhanced sensitivity of ***CaOSR1***-OX transgenic Arabidopsis lines to ABA**. **(A)** Germination rates of *CaOSR1*-OX mutants and wild-type (WT) plants on 0.5 × Murashige and Skoog (MS) medium supplemented with various concentrations of ABA. **(B,C)** Seedling development of *CaOSR1*-OX mutants and wild-type plants exposed to ABA. The numbers of seedlings in each line with expanded cotyledons were counted **(C)** and representative photographs were taken 5 days after plating **(B)**. Data represent the mean ± standard error of three independent experiments, each evaluating 36 seeds. **(D,E)** Root elongation of wild-type and transgenic lines exposed to ABA. The root lengths of each plant were measured 8 days after sowing **(E)** and representative images were taken **(D)**. Data represent the mean ± standard error of three independent experiments, each evaluating 36 seeds. Different letters indicate significant differences between wild-type and transgenic lines (*P* < 0.05; ANOVA followed by Fisher's LSD test).

**Figure 5:**
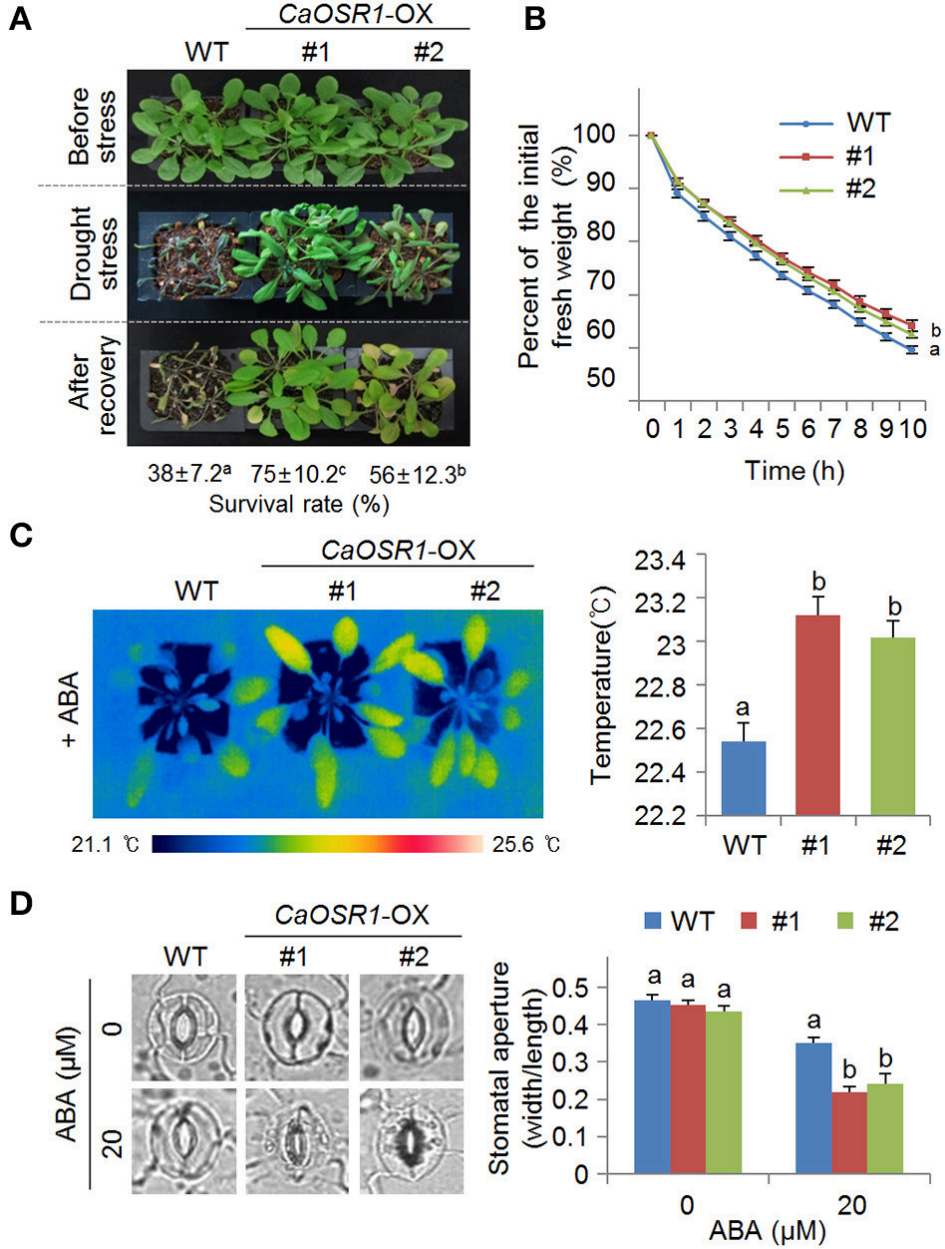
**Enhanced tolerance of ***CaOSR1-OX*** transgenic Arabidopsis lines to drought stress**. **(A)** Drought tolerance of *CaOSR1*-OX transgenic plants. Three-week-old wild-type (WT) and transgenic plants were subjected to drought stress by withholding watering for 9 days and rewatering for 2 days. Survival rates of plants after rewatering. Data represent the mean ± standard error of three independent experiments, each evaluating 20 plants. **(B)** Transpirational water loss from the leaves of wild-type and transgenic plants at various time points after detachment of leaves. Data represent the mean ± standard error of three independent experiments, each evaluating 50 leaves. **(C)** Increased leaf temperatures of *CaOSR1*-OX plants exposed to ABA treatment. Data represent the mean ± standard error of three independent experiments, each evaluating 10 plants. **(D)** Stomatal apertures in *CaOSR1*-OX transgenic and wild-type plants treated with 0 and 20 μM ABA. Leaf peels were harvested from 3-week-old plants and stomatal apertures were measured under a microscope. Data represent the mean ± standard error of three independent experiments, each evaluating 20 plants. Different letters indicate significant differences between wild-type and transgenic lines (*P* < 0.05; ANOVA followed by Fisher's LSD test).

We further studied the function of CaOSR1 in osmotic stress responses by generating transgenic Arabidopsis plants that overexpressed the *CaOSR1* gene under the control of the cauliflower mosaic virus (CaMV) 35S promoter. We obtained two independent T_3_ transgenic lines (*CaOSR1*-OX #1 and *CaOSR1*-OX #2) showing relatively high expression of *CaOSR1* (Supplementary Figure 3), and we used these two lines in our phenotypic analyses. Under normal growth conditions, we observed no significant differences in the growth of wild-type and *CaOSR1*-OX plants (Figures 4, 5). The primary function of ABA is defense response to abiotic stress; moreover, ABA signaling overlaps with the defense signaling response to abiotic stress (Zhu, 2002). To examine ABA sensitivity during germination, we sowed seeds on MS medium supplemented with various concentrations of ABA (0.0, 0.5, and 1.0 μM). In the absence of ABA, we determined no significant difference in germination rates between wild-type and *CaOSR1*-OX seeds. However, in the presence of ABA, the germination rate of *CaOSR1*-OX seeds was lower than that of wild-type seeds (Figure 4A). Next, we determined the rates of cotyledon greening 5 days after sowing and the root lengths 8 days after sowing. We found that treatment with increasing concentrations of ABA resulted in decreased rates of cotyledon greening and reduced root lengths; these effects were observed with ABA concentrations as low as 0.5 μM (Figures 4B–E). As predicted, *CaOSR1*-OX plants exhibited an ABA-hypersensitive phenotype. In the presence of ABA, the rate of cotyledon greening was significantly lower in *CaOSR1*-OX seedlings than in wild-type seedlings (Figures 4B,C); moreover, root elongation of *CaOSR1*-OX seedlings was significantly inhibited (Figures 4D,E). Our results indicate that ectopic expression of *CaOSR1* confers ABA hypersensitivity in Arabidopsis.

### Reduced sensitivity of *CaOSR1*-OX transgenic plants to high salinity and osmotic stress

The expression of *CaOSR1* in pepper leaves was induced by NaCl treatment (Figure 2C), and therefore we predicted that overexpression of this gene alters the response to high salt stress. In the absence of NaCl, we determined no significant differences in germination rates between wild-type and transgenic seeds. However, in the presence of 100 and 150 mM NaCl, the germination rates of *CaOSR1*-OX seeds were significantly higher than those of wild-type seeds (Supplementary Figure 4A). Next, we assessed the seedling growth and development of wild-type and *CaOSR1*-OX plants in the presence of NaCl. We found that *CaOSR1*-OX transgenic plants showed reduced sensitivity to high salinity stress at the seedling stage (Supplementary Figures 4B–E).

*CaOSR1*-OX plants displayed an ABA-hypersensitive phenotype during the seed germination and seedling growth stages (Figure 4), and therefore we postulated that the *CaOSR1*-OX gene functions in the defense response to osmotic stress (Supplementary Figure 5). To test this hypothesis, we first assessed the germination rates in wild-type and transgenic plants after treatment with various concentrations of mannitol. After exposure to 300 or 400 mM mannitol, the germination rate of *CaOSR1*-OX seeds was significantly higher than that of wild-type seeds (Supplementary Figure 5A). Next, we determined the rates of cotyledon greening 5 days after sowing and the root lengths 8 days after sowing (Supplementary Figures 5B–E). We found that after exposure to mannitol, the rate of cotyledon greening and root elongation was significantly higher in *CaOSR1*-OX seedlings than in wild-type seedlings (Supplementary Figures 5B–E). As predicted, our results indicate that *CaOSR1* functions as a regulator of the osmotic stress response.

### Enhanced tolerance of *CaOSR1*-OX transgenic plants to drought stress

*CaOSR1*-OX plants showed less sensitive phenotypes to mannitol- and salt-induced osmotic stresses during the seed germination and seedling growth stages, and therefore we investigated the drought tolerance of these transgenic plants (Figure 5). When grown under well-watered conditions, we observed no phenotypic differences between wild-type and *CaOSR1*-OX plants (Figure 5A, upper panel). However, when we subjected plants to drought stress by withholding watering for 9 days, transgenic plants displayed a less wilted phenotype than wild-type plants (Figure 5A, middle panel). After recovery by rewatering for 2 days, the survival rates of *CaOSR1*-OX lines #1 and #2 were 75 and 56%, respectively, whereas that of wild-type plants was only 38% (Figure 5A, lower panel). Next, we compared the fresh weight of detached rosette leaves to monitor the transpirational water loss and thus determine whether the drought-tolerant phenotype displayed by *CaOSR1*-OX plants was derived from an altered transpiration rate (Figure 5B). We found that the transpiration rate was lower in the leaves of *CaOSR1*-OX plants than in the leaves of wild-type plants, implying that the drought-tolerant phenotype was derived from increased water retention.

Generally, drought tolerance is determined by at least two cellular or molecular parameters. Previous studies have used measurements of leaf temperature and stomatal aperture to establish that ABA hypersensitivity leads to enhanced drought tolerance (Cheong et al., 2007; Lim et al., 2015b; Park S. Y. et al., 2015). Other studies have revealed correlations between drought tolerance and low or high levels of stress-related gene expression; these correlations may lead to increased or decreased drought tolerance, respectively (Gonzalez-Guzman et al., 2012; Park C. et al., 2015). To determine whether the enhanced drought tolerance of *CaOSR1*-OX plants is associated with increased ABA sensitivity, we measured the leaf temperature and stomatal aperture. We found that after ABA treatment, the leaf temperatures were significantly higher in *CaOSR1*-OX plants than in wild-type plants (Figure 5C), implying that the CaOSR1 protein plays a key role in ABA-mediated stomatal closure. In the absence of ABA, we determined no significant differences in leaf temperature and stomatal aperture between the leaves of wild-type and transgenic plants (Supplementary Figure 2B and Figure 5D). However, after exposure to 20 μM ABA, the stomatal apertures in the leaves of *CaOSR1*-OX plants were significantly smaller than those in the leaves of wild-type plants (Figure 5D).

**Figure 6:**
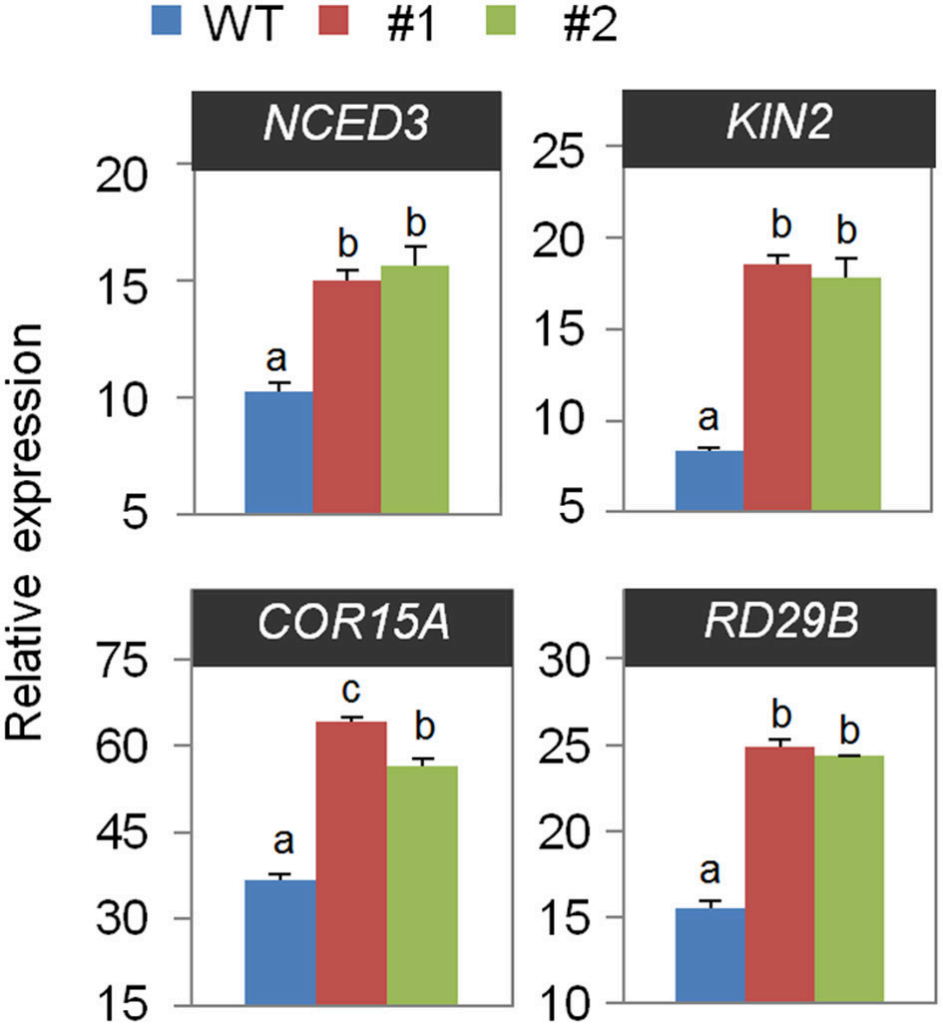
**Quantitative reverse transcription polymerase chain reaction (qRT-PCR) analysis of drought-inducible genes in the ***CaOSR1-OX*** mutant exposed to drought stress at 6 h after detachment**. The relative expression levels (ΔΔCT) of each gene were normalized to the geometric mean of *Actin8* as an internal control gene. Data represent the mean ± standard error of three independent experiments. Different letters indicate significant differences between wild-type and transgenic lines (*P* < 0.05; ANOVA followed by Fisher's LSD test).

Finally, we performed quantitative reverse transcription-polymerase chain reaction (qRT-PCR) analysis in wild-type and *CaOSR1*-OX plants to investigate the function of *CaOSR1* in controlling the expression of stress-responsive genes under drought conditions (Figure 6). In general, the levels of ABA in plant tissues increase under drought stress conditions, and this induces the expression of many stress-related genes, including *NCED3, KIN2, COR15A*, and *RD29B*, After 6 h of drought stress treatment, we determined significantly higher expression levels of these genes in *CaOSR1*-OX plants than in wild-type plants, implying that conferred expression of *CaOSR1* affects the expression levels of stress-related genes. Taken together, our results confirm our hypothesis that the CaOSR1 protein functions as a positive regulator of multiple osmotic stresses in plants.

## Discussion

In the present study, we identified and functionally characterized CaOSR1, which functions as a positive regulator in the osmotic stress response via ABA-mediated signaling. Under water-deficit conditions, expression of *CaOSR1* in pepper and Arabidopsis plants resulted in altered phenotypes.

The function of specific proteins in plant cells can be predicted by homology search analysis with other known proteins. Using the deduced amino acid sequence of *CaOSR1* gene as query, a BLASTP search at NCBI revealed that CaOSR1 is highly close to low-temperature induced (LTI) protein 65, but uncharacterized, from several plant species. Most of these proteins have distinctly two conserved domains: acidic domain and CAP160 domain. Of them, we initially focused on Arabidopsis RD29B, alternately named LTI65, even though CaOSR1 has low homology to RD29B. It is well-known that *RD29B* and its homologous gene *RD29A* are induced by cold, drought, NaCl, and ABA treatments (Yamaguchi-Shinozaki and Shinozaki, 1993; Msanne et al., 2011). Promoter sequences of *RD29A* and *RD29B* genes have different number of Dehydration-Responsive Element (DRE) and ABA-Responsive Element (ABRE): several DRE and one ABRE in *RD29A* and several ABRE and one DRE in RD29B (Msanne et al., 2011). Similarly to *RD29B* promoter, 2kb-upstream sequence of *CaOSR1* gene contains one core motif of dehydration-responsive element/C-repeat (DRE/CRT; GCCGAC) and 7 ABREs (CACGT). This feature may contribute to induction of CaOSR1 in response to ABA, drought, and salt treatments as shown in Figure 2. Considered primary structure of RD29B and RD29A, we found that CAP160 domain is found in RD29B, not RD29A. Although internal sequence repeats that are shown in CaOSR1 are found only in RD29A, we predicted that CaOSR1 can be orthologous protein of Arabidopsis RD29B and contribute to mitigating abiotic stress.

Intriguingly, CaOSR1 showed different pattern of subcellular localization compared with RD29B: CaOSR1-GFP fusion proteins are localized in the nucleus (Figure 2D), while RD29B targets to the cytoplasm (Msanne et al., 2011). Information on protein subcellular localization is very crucial because it provides clue to understand biological function of protein. Considered this difference and low sequence homology between CaOSR1 and RD29B, we cannot rule out the possibility that CaOSR1 can have alternative biological functions in pepper plants, compared with RD29B. Next, we focused on CAP160 domain which is first reported in Spinach CAP160 protein (Kaye et al., 1998). Similarly to *RD29B, CAP160* gene is induced by drought stress as well as low temperature exposure and its encoded protein localized to the cytoplasm. However, sequence homology between the two proteins is low (25.9% identity/36.7% similarity) and this pattern is also shown between CaOSR1 and CAP160 (22.9% identity/33.3% similarity). Functional role of RD29B and CAP160 remains unclear and shared sequence homology between the proteins is very limited, which imply that CaOSR1 may be a novel stress-responsive protein in pepper plant.

To examine the role of *CaOSR1*, we conducted VIGS and overexpression-based genetic analysis for loss-of function and gain-of function in pepper and Arabidopsis, respectively. We found that *CaOSR1*-silenced pepper plants showed increased drought susceptibility; this was characterized by decreased water retention capacity, indicating that compromised expression of *CaOSR1* impedes stomatal closure. In contrast to *CaOSR1*-silenced pepper plants, *CaOSR1*-OX Arabidopsis plants exhibited enhanced tolerance to drought stress via ABA-mediated signaling. In addition, we demonstrated that *CaOSR1*-OX plants were hypersensitive to ABA, implying that *CaOSR1* regulates osmotic stress tolerance via ABA-mediated cell signaling. We further observed no phenotypic differences between *CaOSR1*-silenced pepper plants and control plants after exposure to NaCl (data not shown). On the other hand, *CaOSR1*-OX plants showed hyposensitivity to high salt stress during the seed germination and seedling growth stages, implying that *CaOSR1* acts as a positive regulator of the high salinity stress response. The observed phenotypic discrepancy between *CaOSR1*-silenced pepper plants and *CaOSR1*-OX transgenic Arabidopsis plants is presumably derived either from the use of different species or from the functions of *CaOSR1* that distinguish it from Arabidopsis genes in terms of response to high salinity.

Based on the effect of the expression level of *CaOSR1* on the sensitivity to ABA, we examined whether *CaOSR1* affects the expression of the *NCED3* gene, which encodes the rate-limiting ABA biosynthesis enzyme in Arabidopsis (Iuchi et al., 2001; Tan et al., 2003). Under optimal plant growth conditions, we determined no significant difference in the expression level of *NCED3* between *CaOSR1*-OX and wild-type plants. However, after 6 h of drought stress treatment, we determined significantly higher expression levels of this gene in *CaOSR1*-OX plants than in wild-type plants (Figure 6). Furthermore, *CaOSR1*-OX plants showed higher accumulation levels of the stress-responsive marker genes *RD29B, KIN2*, and *COR15A*, which are associated with abiotic tolerance via ABA-dependent and ABA-independent pathways, respectively (Artus et al., 1996; Uno et al., 2000; Hoth et al., 2002). Our results imply that CaOSR1 acts upstream of these genes in the drought stress response. In addition, the enhanced expression levels of abiotic stress marker genes may reflect the increased ABA sensitivity of *CaOSR1*-OX plants. Nevertheless, the increased or decreased expression levels of stress-related genes do not fully explain the altered phenotypes displayed by *CaOSR1*-OX plants in response to ABA and osmotic stress treatments.

In conclusion, we have shown that CaOSR1 functions as a positive regulator of the osmotic stress response in plants. *CaOSR1*-OX plants exhibited an ABA-hypersensitive phenotype, and this was characterized by increased ABA-induced stomatal closure and enhanced expression levels of ABA-mediated stress-responsive genes. Our findings provide a valuable insight into the osmotic stress response in plants. Nevertheless, the precise mechanism whereby CaOSR1 serves as a positive component of osmotic stress responses remains unclear. Further studies based on genetic and molecular analysis of the upstream and downstream regions of the *CaOSR1* gene will help to clarify the role of CaOSR1 in the osmotic stress response.

## Author contributions

CP and CL performed experiments and analyzed the results. SL designed the experiments and wrote the manuscript.

### Conflict of interest statement

The authors declare that the research was conducted in the absence of any commercial or financial relationships that could be construed as a potential conflict of interest.

## Supplementary material

Supplementary Table 1 **Sequences of primers used in this study**.

Supplementary Figure 1 **Multiple alignment of amino acids in the CaOSR1 protein and its homologous proteins was performed using ClustalW2**.

Supplementary Figure 2 **(A)** Mean leaf temperatures of *CaOSR1*-silenced and vector control pepper plants. **(B)** Mean leaf temperatures of *CaOSR1*-OX and wild-type Arabidopsis plants. Data represent the mean ± standard error of three independent experiments, each evaluating 10 plants.

Supplementary Figure 3 **Reverse transcription-polymerase chain reaction (RT-PCR) analysis of *CaOSR1* expression in wild-type (WT) and *CaOSR1*-OX transgenic lines**. *Actin8* was used as an internal control gene.

Supplementary Figure 4 **Reduced sensitivity of *CaOSR1*-OX transgenic Arabidopsis lines to high salt stress during germination and seedling development. (A)** Germination rates of *CaOSR1*-OX mutants and wild-type (WT) plants on 0.5 × MS medium supplemented with various concentrations of NaCl. Data represent the mean ± standard error of three independent experiments, each evaluating 36 seeds. **(B,C)** Seedling development of *CaOSR1*-OX mutants and wild-type plants exposed to NaCl. The numbers of seedlings in each line with expanded cotyledons were counted **(C)** and representative photographs were taken 5 days after plating **(B)**. Data represent the mean ± standard error of three independent experiments, each evaluating 36 seeds. **(D,E)** Root elongation of wild-type and transgenic lines exposed to NaCl. The root lengths of each plant were measured 8 days after sowing **(E)** and representative images were taken **(D)**. Data represent the mean ± standard error of three independent experiments. Different letters indicate significant differences between wild-type and transgenic lines (*P* < 0.05; ANOVA followed by Fisher's LSD test).

Supplementary Figure 5 **Reduced sensitivity of *CaOSR1*-OX transgenic Arabidopsis lines to osmotic stress during germination and seedling development. (A)** Germination rates of *CaOSR1*-OX mutants and wild-type (WT) plants on 0.5 × MS medium supplemented with various concentrations of mannitol. Data represent the mean ± standard error of three independent experiments, each evaluating 36 seeds. **(B,C)** Seedling development of *CaOSR1*-OX mutants and wild-type plants exposed to mannitol. The numbers of seedlings in each line with expanded cotyledons were counted **(C)** and representative photographs were taken 5 days after plating **(B)**. Data represent the mean ± standard error of three independent experiments, each evaluating 36 seeds. **(D,E)** Root elongation of wild-type and transgenic lines exposed to mannitol. The root lengths of each plant were measured 8 days after sowing **(E)** and representative images were taken **(D)**. Data represent the mean ± standard error of three independent experiments, each evaluating 36 seeds. Different letters indicate significant differences between wild-type and transgenic lines (*P* < 0.05; ANOVA followed by Fisher's LSD test).

## References

[B1] Ambrosone A. Batelli G. Nurcato R. Aurilia V. Punzo P. Bangarusamy D. K.. (2015). The arabidopsis RNA-binding protein AtRGGA regulates tolerance to salt and drought stress. Plant Physiol. 168, 292–306. 10.1104/pp.114.255802 25783413 PMC4424017

[B2] Artus N. N. Uemura M. Steponkus P. L. Gilmour S. J. Lin C. Thomashow M. F. (1996). Constitutive expression of the cold-regulated *Arabidopsis thaliana COR15a* gene affects both chloroplast and protoplast freezing tolerance. Proc. Natl. Acad. Sci. U.S.A. 93, 13404–13409. 11038526 10.1073/pnas.93.23.13404 PMC24106

[B3] Cheong Y. H. Pandey G. K. Grant J. J. Batistic O. Li L. Kim B. G.. (2007). Two calcineurin B-like calcium sensors, interacting with protein kinase CIPK23, regulate leaf transpiration and root potassium uptake in Arabidopsis. Plant J. 52, 223–239. 10.1111/j.1365-313X.2007.03236.x 17922773

[B4] Ding Y. Li H. Zhang X. Xie Q. Gong Z. Yang S. (2015). OST1 kinase modulates freezing tolerance by enhancing ICE1 stability in Arabidopsis. Dev. Cell 32, 278–289. 10.1016/j.devcel.2014.12.023 25669882

[B5] Dubois M. Van den Broeck L. Claeys H. Van Vlierberghe K. Matsui M. Inze D. (2015). The ETHYLENE RESPONSE FACTORs ERF6 and ERF11 antagonistically regulate mannitol-induced growth inhibition in Arabidopsis. Plant Physiol. 169, 166–179. 10.1104/pp.15.00335 25995327 PMC4577380

[B6] Geiger D. Scherzer S. Mumm P. Stange A. Marten I. Bauer H.. (2009). Activity of guard cell anion channel SLAC1 is controlled by drought-stress signaling kinase-phosphatase pair. Proc. Natl. Acad. Sci. U.S.A. 106, 21425–21430. 10.1073/pnas.0912021106 19955405 PMC2795561

[B7] Goda H. Sasaki E. Akiyama K. Maruyama-Nakashita A. Nakabayashi K. Li W.. (2008). The AtGenExpress hormone and chemical treatment data set: experimental design, data evaluation, model data analysis and data access. Plant J. 55, 526–542. 10.1111/j.0960-7412.2008.03510.x 18419781

[B8] Gonzalez-Guzman M. Pizzio G. A. Antoni R. Vera-Sirera F. Merilo E. Bassel G. W.. (2012). Arabidopsis PYR/PYL/RCAR receptors play a major role in quantitative regulation of stomatal aperture and transcriptional response to abscisic acid. Plant Cell 24, 2483–2496. 10.1105/tpc.112.098574 22739828 PMC3406898

[B9] Grondin A. Rodrigues O. Verdoucq L. Merlot S. Leonhardt N. Maurel C. (2015). Aquaporins contribute to ABA-triggered stomatal closure through OST1-mediated phosphorylation. Plant Cell 27, 1945–1954. 10.1105/tpc.15.00421 26163575 PMC4531361

[B10] Hoth S. Morgante M. Sanchez J. P. Hanafey M. K. Tingey S. V. Chua N. H. (2002). Genome-wide gene expression profiling in *Arabidopsis thaliana* reveals new targets of abscisic acid and largely impaired gene regulation in the abi1-1 mutant. J. Cell Sci. 115(Pt 24), 4891–4900. 10.1242/jcs.00175 12432076

[B11] Iuchi S. Kobayashi M. Taji T. Naramoto M. Seki M. Kato T.. (2001). Regulation of drought tolerance by gene manipulation of 9-*cis*-epoxycarotenoid dioxygenase, a key enzyme in abscisic acid biosynthesis in *Arabidopsis*. Plant J. 27, 325–333. 10.1046/j.1365-313x.2001.01096.x 11532178

[B12] Jakab G. Ton J. Flors V. Zimmerli L. Metraux J. P. Mauch-Mani B. (2005). Enhancing Arabidopsis salt and drought stress tolerance by chemical priming for its abscisic acid responses. Plant Physiol. 139, 267–274. 10.1104/pp.105.065698 16113213 PMC1203376

[B13] Kaye C. Neven L. Hofig A. Li Q. B. Haskell D. Guy C. (1998). Characterization of a gene for spinach CAP160 and expression of two spinach cold-acclimation proteins in tobacco. Plant Physiol. 116, 1367–1377. 9536054 10.1104/pp.116.4.1367 PMC35044

[B14] Kim S. Kang J. Y. Cho D. I. Park J. H. Kim S. Y. (2004). ABF2, an ABRE-binding bZIP factor, is an essential component of glucose signaling and its overexpression affects multiple stress tolerance. Plant J. 40, 75–87. 10.1111/j.1365-313X.2004.02192.x 15361142

[B15] Lee C. M. Thomashow M. F. (2012). Photoperiodic regulation of the C-repeat binding factor (CBF) cold acclimation pathway and freezing tolerance in *Arabidopsis thaliana*. Proc. Natl. Acad. Sci. U.S.A. 109, 15054–15059. 10.1073/pnas.1211295109 22927419 PMC3443188

[B16] Lee S. C. Hwang I. S. Choi H. W. Hwang B. K. (2008). Involvement of the pepper antimicrobial protein *CaAMP1* gene in broad spectrum disease resistance. Plant Physiol. 148, 1004–1020. 10.1104/pp.108.123836 18676663 PMC2556820

[B17] Lee S. C. Lan W. Buchanan B. B. Luan S. (2009). A protein kinase-phosphatase pair interacts with an ion channel to regulate ABA signaling in plant guard cells. Proc. Natl. Acad. Sci. U.S.A. 106, 21419–21424. 10.1073/pnas.0910601106 19955427 PMC2795491

[B18] Lee S. C. Lim C. W. Lan W. He K. Luan S. (2013). ABA signaling in guard cells entails a dynamic protein-protein interaction relay from the PYL-RCAR family receptors to ion channels. Mol. Plant 6, 528–538. 10.1093/mp/sss078 22935148

[B19] Lee S. C. Luan S. (2012). ABA signal transduction at the crossroad of biotic and abiotic stress responses. Plant Cell Environ. 35, 53–60. 10.1111/j.1365-3040.2011.02426.x 21923759

[B20] Lee S. J. Kang J. Y. Park H. J. Kim M. D. Bae M. S. Choi H. I.. (2010). DREB2C interacts with ABF2, a bZIP protein regulating abscisic acid-responsive gene expression, and its overexpression affects abscisic acid sensitivity. Plant Physiol. 153, 716–727. 10.1104/pp.110.154617 20395451 PMC2879808

[B21] Li Z. Zhang L. Yu Y. Quan R. Zhang Z. Zhang H.. (2011). The ethylene response factor AtERF11 that is transcriptionally modulated by the bZIP transcription factor HY5 is a crucial repressor for ethylene biosynthesis in Arabidopsis. Plant J. 68, 88–99. 10.1111/j.1365-313X.2011.04670.x 21645149

[B22] Lim C. W. Baek W. Jung J. Kim J. H. Lee S. C. (2015a). Function of ABA in stomatal defense against biotic and drought stresses. Int. J. Mol. Sci. 16, 15251–15270. 10.3390/ijms160715251 26154766 PMC4519898

[B23] Lim C. W. Han S. W. Hwang I. S. Kim D. S. Hwang B. K. Lee S. C. (2015b). The pepper lipoxygenase CaLOX1 plays a role in osmotic, drought and high salinity stress response. Plant Cell Physiol. 56, 930–942. 10.1093/pcp/pcv020 25657344

[B24] Lim C. W. Lee S. C. (2015). Arabidopsis abscisic acid receptors play an important role in disease resistance. Plant Mol. Biol. 88, 313–324. 10.1007/s11103-015-0330-1 25969135

[B25] Lim S. Baek W. Lee S. C. (2014). Identification and functional roles of CaDIN1 in abscisic acid signaling and drought sensitivity. Plant Mol. Biol. 86, 513–525. 10.1007/s11103-014-0242-5 25149469

[B26] Livak K. J. Schmittgen T. D. (2001). Analysis of relative gene expression data using real-time quantitative PCR and the 2(-Delta Delta C(T)) Method. Methods 25, 402–408. 10.1006/meth.2001.1262 11846609

[B27] Mizuno T. Yamashino T. (2008). Comparative transcriptome of diurnally oscillating genes and hormone-responsive genes in *Arabidopsis thaliana*: insight into circadian clock-controlled daily responses to common ambient stresses in plants. Plant Cell Physiol. 49, 481–487. 10.1093/pcp/pcn008 18202002

[B28] Msanne J. Lin J. Stone J. M. Awada T. (2011). Characterization of abiotic stress-responsive *Arabidopsis thaliana* RD29A and RD29B genes and evaluation of transgenes. Planta 234, 97–107. 10.1007/s00425-011-1387-y 21374086

[B29] Park C. Lim C. W. Baek W. Lee S. C. (2015). RING type E3 ligase CaAIR1 in pepper acts in the regulation of ABA signaling and drought stress response. Plant Cell Physiol. 56, 1808–1819. 10.1093/pcp/pcv103 26169196

[B30] Park S. Y. Peterson F. C. Mosquna A. Yao J. Volkman B. F. Cutler S. R. (2015). Agrochemical control of plant water use using engineered abscisic acid receptors. Nature 520, 545–548. 10.1038/nature14123 25652827

[B31] Sengupta S. Majumder A. L. (2010). *Porteresia coarctata* (Roxb.) Tateoka, a wild rice: a potential model for studying salt-stress biology in rice. Plant Cell Environ. 33, 526–542. 10.1111/j.1365-3040.2009.02054.x 19843254

[B32] Shinozaki K. Yamaguchi-Shinozaki K. (2000). Molecular responses to dehydration and low temperature: differences and cross-talk between two stress signaling pathways. Curr. Opin. Plant Biol. 3, 217–223. 10.1016/S1369-5266(00)80068-0 10837265

[B33] Shinozaki K. Yamaguchi-Shinozaki K. (2007). Gene networks involved in drought stress response and tolerance. J. Exp. Bot. 58, 221–227. 10.1093/jxb/erl164 17075077

[B34] Tan B. C. Joseph L. M. Deng W. T. Liu L. Li Q. B. Cline K.. (2003). Molecular characterization of the Arabidopsis 9-cis epoxycarotenoid dioxygenase gene family. Plant J. 35, 44–56. 10.1046/j.1365-313X.2003.01786.x 12834401

[B35] Umezawa T. Sugiyama N. Takahashi F. Anderson J. C. Ishihama Y. Peck S. C.. (2013). Genetics and phosphoproteomics reveal a protein phosphorylation network in the abscisic acid signaling pathway in *Arabidopsis thaliana*. Sci. Signal. 6:rs8. 10.1126/scisignal.2003509 23572148

[B36] Uno Y. Furihata T. Abe H. Yoshida R. Shinozaki K. Yamaguchi-Shinozaki K. (2000). Arabidopsis basic leucine zipper transcription factors involved in an abscisic acid-dependent signal transduction pathway under drought and high-salinity conditions. Proc. Natl. Acad. Sci. U.S.A. 97, 11632–11637. 10.1073/pnas.190309197 11005831 PMC17252

[B37] Vlad F. Rubio S. Rodrigues A. Sirichandra C. Belin C. Robert N.. (2009). Protein phosphatases 2C regulate the activation of the Snf1-related kinase OST1 by abscisic acid in Arabidopsis. Plant Cell 21, 3170–3184. 10.1105/tpc.109.069179 19855047 PMC2782292

[B38] Yamaguchi-Shinozaki K. Shinozaki K. (1993). Characterization of the expression of a desiccation-responsive rd29 gene of *Arabidopsis thaliana* and analysis of its promoter in transgenic plants. Mol. Gen. Genet. 236, 331–340. 8437577 10.1007/BF00277130

[B39] Yoshida T. Fujita Y. Maruyama K. Mogami J. Todaka D. Shinozaki K.. (2015). Four Arabidopsis AREB/ABF transcription factors function predominantly in gene expression downstream of SnRK2 kinases in abscisic acid signalling in response to osmotic stress. Plant Cell Environ. 38, 35–49. 10.1111/pce.12351 24738645 PMC4302978

[B40] Zhu J. K. (2002). Salt and drought stress signal transduction in plants. Annu. Rev. Plant Biol. 53, 247–273. 10.1146/annurev.arplant.53.091401.143329 12221975 PMC3128348

[B41] Zou J. J. Li X. D. Ratnasekera D. Wang C. Liu W. X. Song L. F.. (2015). Arabidopsis CALCIUM-DEPENDENT PROTEIN KINASE8 and CATALASE3 function in abscisic acid-mediated signaling and H_2_O_2_ homeostasis in stomatal guard cells under drought stress. Plant Cell 27, 1445–1460. 10.1105/tpc.15.00144 25966761 PMC4456645

