# Supplementary material for: The Pepper CaOSR1 Protein Regulates the Osmotic Stress Response via Abscisic Acid Signaling
Source: Front Plant Sci. 2016 Jun 24;7:890. doi: 10.3389/fpls.2016.00890 (PMC4919342; doi:10.3389/fpls.2016.00890)
Supplement: Supplementary Table 1 — Sequences of primers used in this study. [file Table1.PDF]

Supplementary Table 1. Sequences of primers used in this study

| Primer name | Primer sequence (5'-3') |
|---|---|
| For cloning | |
| *CaOSR1* | Forward: ATGGAGGCACAACTGCACCGTC |
| | Reverse: TTATTCAACCCTTCCCCCAACA |
| For RT-PCR | |
| *CaOSR1* | Forward: CTCGAGCTATCAGAGCAAAGTC |
| | Reverse: TCTAGAGATCCTGTGTATTGACCAT |
| *CaACT1* | Forward: GACGTGACCTAACTGATAACCTGAT |
| | Reverse: CTCTCAGCACCAATGGTAATAACTT |
| *Actin8* | Forward: CAACTATGTTCTCAGGTATTGCAGA |
| | Reverse: GTCATGGAAACGATGTCTCTTTAGT |
| *NCED3* | Forward: ACATGGAAATCGGAGTTACAGATAG |
| | Reverse: AGAAACAACAAACAAGAAACAGAGC |
| *KIN2* | Forward: TGTTAACTTCGTGAAGGACAAGAC |
| | Reverse: ACAACAACAAGTACGATGAGTACGA |
| *COR15A* | Forward: GATACATTGGGTAAAGAAGCTGAGA |
| | Reverse: ACATGAAGAGAGAGGATATGGATCA |
| *RD29B* | Forward: GTTGAAGAGTCTCCACAATCACTTG |
| | Reverse: ATACAAATCCCCAAACTGAATAACA |
