# Supplementary figures and images for: The Pepper CaOSR1 Protein Regulates the Osmotic Stress Response via Abscisic Acid Signaling
Source: Front Plant Sci. 2016 Jun 24;7:890. doi: 10.3389/fpls.2016.00890 (PMC4919342; doi:10.3389/fpls.2016.00890)

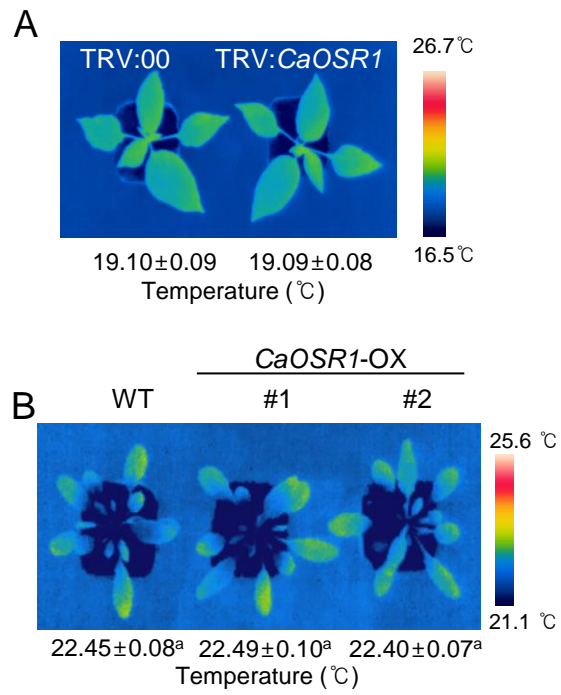

Supplementary Fig. 2 Park et al.

Supplement: Supplementary Figure 2 — (A) Mean leaf temperatures of CaOSR1-silenced and vector control pepper plants. (B) Mean leaf temperatures of CaOSR1-OX and wild-type Arabidopsis plants. Data represent the mean ± standard error of three independent experiments, each evaluating 10 plants. [file Image2.PDF]

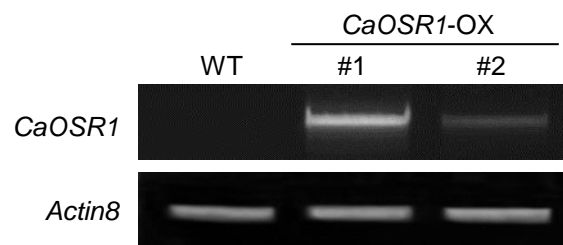

Supplementary Fig. 3 Park et al.

Supplement: Supplementary Figure 3 — Reverse transcription-polymerase chain reaction (RT-PCR) analysis of CaOSR1 expression in wild-type (WT) and CaOSR1-OX transgenic lines. Actin8 was used as an internal control gene. [file Image3.PDF]

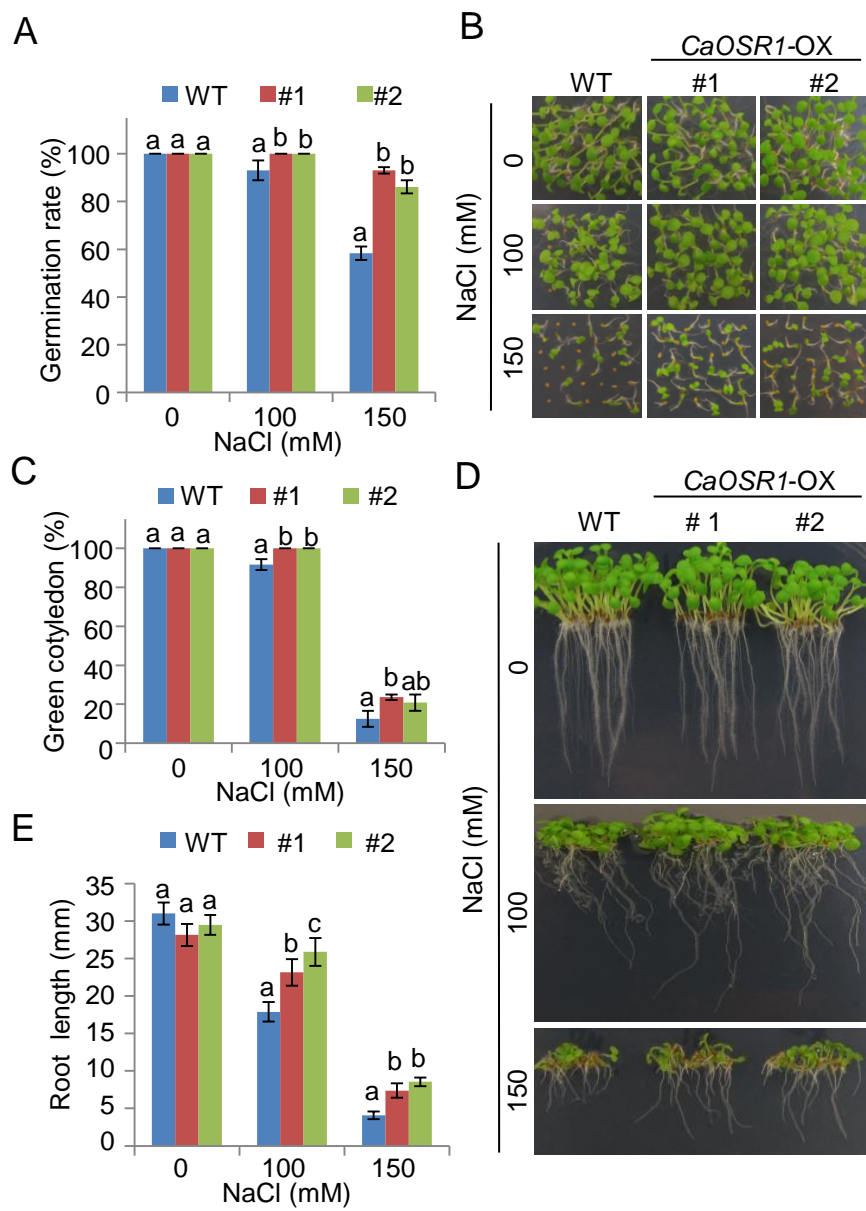

Supplementary Fig. 4 Park et al.

Supplement: Supplementary Figure 4 — Reduced sensitivity of CaOSR1-OX transgenic Arabidopsis lines to high salt stress during germination and seedling development. (A) Germination rates of CaOSR1-OX mutants and wild-type (WT) plants on 0.5 × MS medium supplemented with various concentrations of NaCl. Data represent the mean ± standard error of three independent experiments, each evaluating 36 seeds. (B,C) Seedling development of CaOSR1-OX mutants and wild-type plants exposed to NaCl. The numbers of seedlings in each line with expanded cotyledons were counted (C) and representative photographs were taken 5 days after plating (B). Data represent the mean ± standard error of three independent experiments, each evaluating 36 seeds. (D,E) Root elongation of wild-type and transgenic lines exposed to NaCl. The root lengths of each plant were measured 8 days after sowing (E) and representative images were taken (D). Data represent the mean ± standard error of three independent experiments. Different letters indicate significant differences between wild-type and transgenic lines (P < 0.05; ANOVA followed by Fisher's LSD test). [file Image4.PDF]

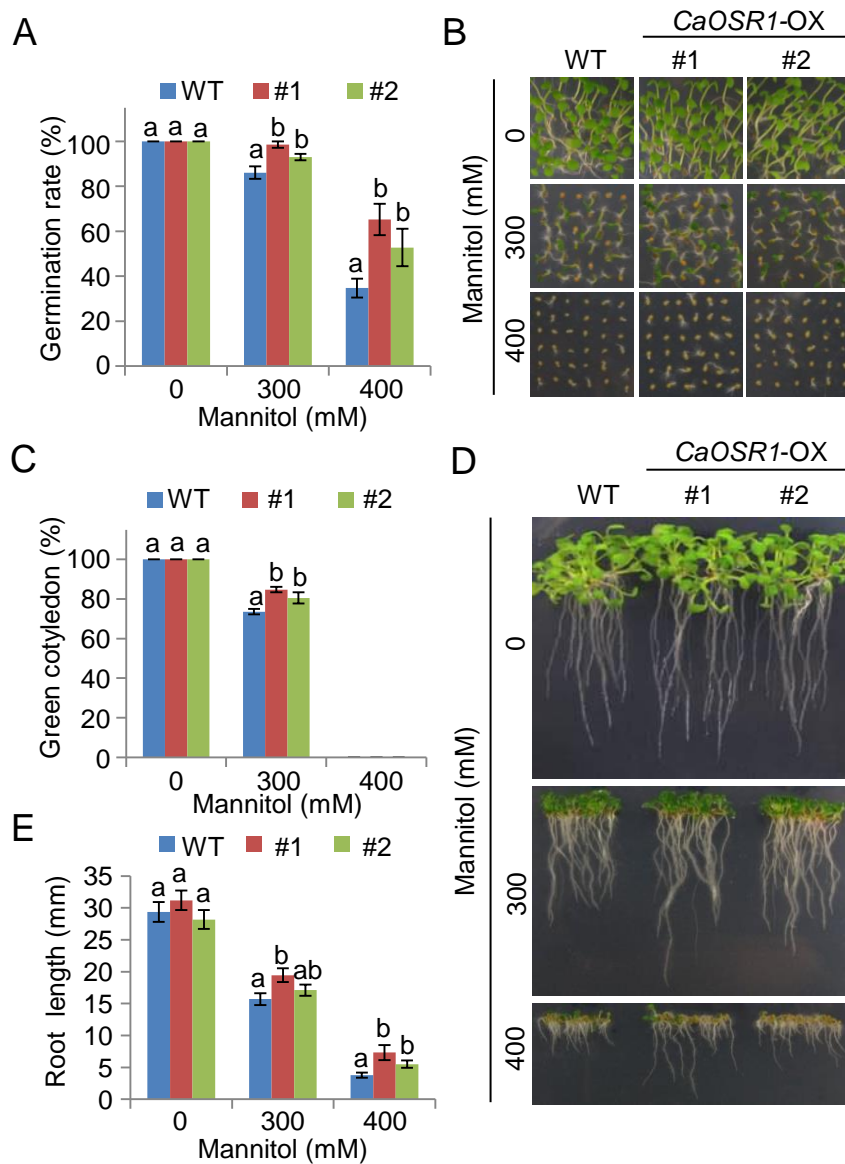

Supplementary Fig. 5 Park et al.

Supplement: Supplementary Figure 5 — Reduced sensitivity of CaOSR1-OX transgenic Arabidopsis lines to osmotic stress during germination and seedling development. (A) Germination rates of CaOSR1-OX mutants and wild-type (WT) plants on 0.5 × MS medium supplemented with various concentrations of mannitol. Data represent the mean ± standard error of three independent experiments, each evaluating 36 seeds. (B,C) Seedling development of CaOSR1-OX mutants and wild-type plants exposed to mannitol. The numbers of seedlings in each line with expanded cotyledons were counted (C) and representative photographs were taken 5 days after plating (B). Data represent the mean ± standard error of three independent experiments, each evaluating 36 seeds. (D,E) Root elongation of wild-type and transgenic lines exposed to mannitol. The root lengths of each plant were measured 8 days after sowing (E) and representative images were taken (D). Data represent the mean ± standard error of three independent experiments, each evaluating 36 seeds. Different letters indicate significant differences between wild-type and transgenic lines (P < 0.05; ANOVA followed by Fisher's LSD test). [file Image5.PDF]
