# Supplementary material for: The Pepper CaOSR1 Protein Regulates the Osmotic Stress Response via Abscisic Acid Signaling
Source: Front Plant Sci. 2016 Jun 24;7:890. doi: 10.3389/fpls.2016.00890 (PMC4919342; doi:10.3389/fpls.2016.00890)
Supplement: Supplementary Figure 1 — Multiple alignment of amino acids in the CaOSR1 protein and its homologous proteins was performed using ClustalW2. [file Image1.PDF]

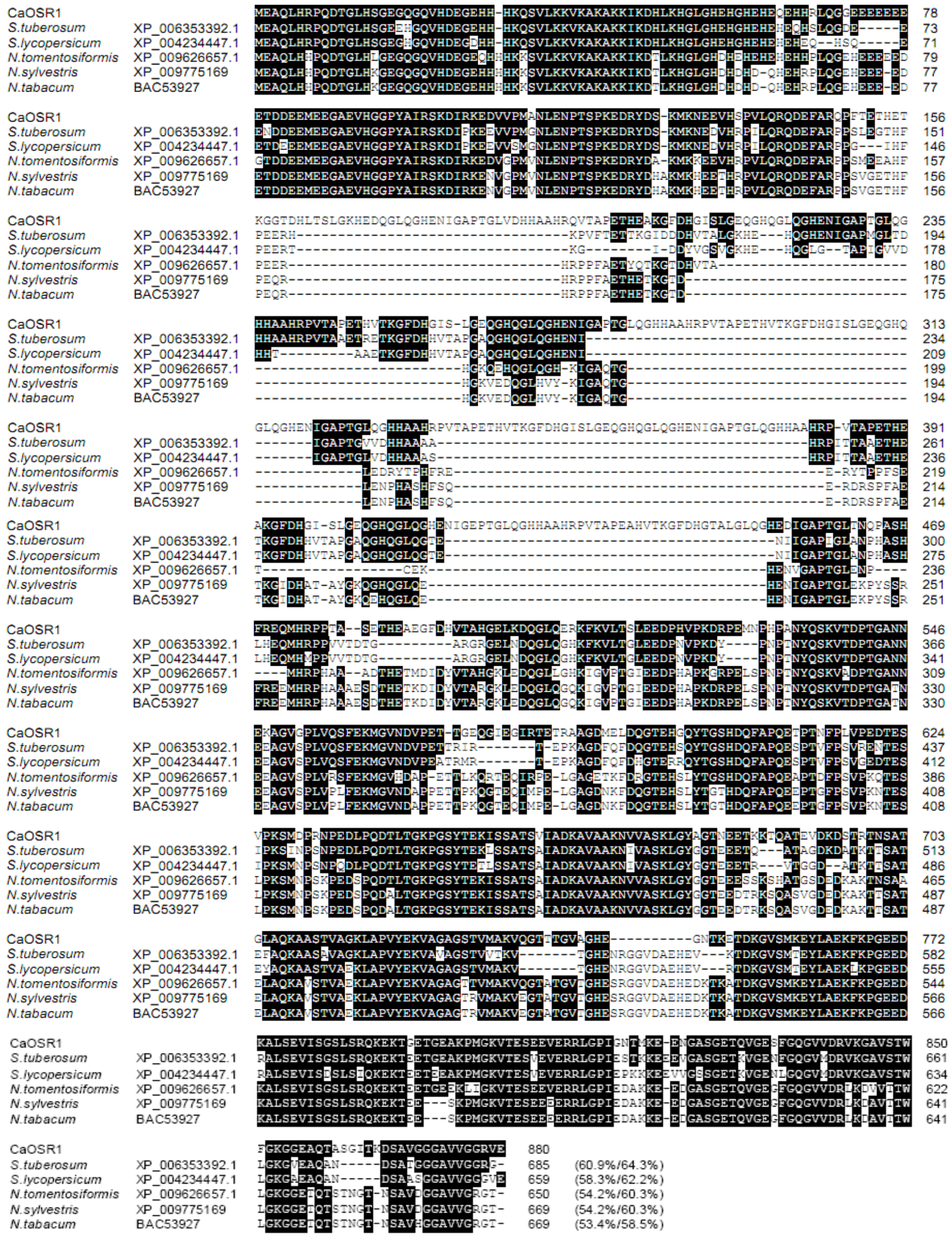

Supplementary Fig. 1 Park et al.
